# Tumor-Associated Macrophages (TAMs) in Cancer: Functional Programs, Metastatic Mechanisms, and Therapeutic Targeting

**DOI:** 10.3390/cancers18091410

**Published:** 2026-04-29

**Authors:** Kisho Ono, Fatemeh Momen-Heravi

**Affiliations:** 1 Department of Orofacial Sciences, School of Dentistry, University of California San Francisco, San Francisco, CA 94143, USA; de20012@s.okayama-u.ac.jp; 2 Cancer Biology and Immunology Laboratory, School of Dentistry, University of California San Francisco, San Francisco, CA 94143, USA; 3 Department of Oral and Maxillofacial Surgery, Faculty of Medicine, Dentistry and Pharmaceutical Sciences, Okayama University, Okayama 700-8558, Japan; 4 Helen Diller Family Comprehensive Cancer Center, University of California San Francisco, San Francisco, CA 94143, USA; 5 Division of Periodontology, University of California San Francisco, San Francisco, CA 94143, USA; 6 ImmunoX Initiative, University of California San Francisco, San Francisco, CA 94143, USA; 7 The Eli and Edythe Broad Center of Regeneration Medicine and Stem Cell Research, San Francisco, CA 94143, USA

**Keywords:** tumor-associated macrophages, breast cancer, metastasis, TMEM, intravasation, immune suppression, CSF1R, CCL2/CCR2, TIE2, lymphangiogenesis

## Abstract

Tumor-associated macrophages (TAMs) are among the most abundant immune cell populations in breast cancer and have emerged as central regulators of tumor progression, metastatic dissemination, immune evasion, and therapeutic resistance. While TAMs were historically described using a simplified M1/M2 polarization framework, accumulating evidence indicates that TAMs in breast cancer comprise a continuum of phenotypic and functional states shaped by ontogeny (tissue-resident vs monocyte-derived), spatial localization (including hypoxic, perivascular, and invasive niches), tumor-intrinsic programs, and therapy-induced selective pressures. In breast cancer, mechanistic studies integrating lineage tracing, intravital imaging, single-cell and spatial profiling, and clinical analyses have established that TAMs actively coordinate rate-limiting steps of the metastatic cascade. These include promotion of angiogenesis and vascular permeability, orchestration of tumor cell invasion and TMEM-mediated intravasation, facilitation of metastatic seeding and niche formation, and suppression of anti-tumor immunity. TAMs also critically influence therapeutic response by modulating chemotherapy efficacy and limiting the activity of immune checkpoint blockade. Therapeutic strategies targeting TAMs in breast cancer have evolved from depletion approaches (CSF1/CSF1R blockade) to inhibition of monocyte recruitment (CCL2/CCR2 axis), functional reprogramming (CD40 agonism, PI3Kγ inhibition), and macrophage-directed checkpoint modulation (CD47–SIRPα axis). Early clinical studies demonstrate clear pharmacodynamic activity but highlight the need for context-specific and combination-based approaches. This review focuses on TAM biology in breast cancer progression and metastasis, synthesizing key mechanistic and translational evidence and proposing a framework in which spatially and functionally defined macrophage states act as rate-limiting regulators of dissemination and therapy response. We further outline principles for rational TAM-targeting strategies that integrate tumor stage, metastatic niche, and treatment context.

## Simple Summary

Macrophages are immune cells that normally support tissue repair and fight infections. In many tumors, macrophages are reprogrammed by cancer cells and the surrounding stroma to support tumor growth and spread. This review summarizes how tumor-associated macrophages can promote breast cancer progression by facilitating tumor cell invasion surrounding tissue, enter blood vessels, and establish metastatic sites in organs such as the lung and bone. We highlight a well-characterized intravasation structure in breast cancer called the Tumor Microenvironment of Metastasis (TMEM), where specialized perivascular macrophages transiently increase vascular permeability, enabling tumor cells to enter the circulation. We also discuss how standard therapies can unintentionally amplify macrophage-dependent dissemination pathways and outline emerging strategies to target specific macrophage programs rather than broadly depleting all macrophages.

## 1. Introduction

Macrophages are evolutionarily ancient cells optimized for tissue surveillance, repair and homeostasis. In breast cancer, these cells—commonly referred to as tumor-associated macrophages (TAMs)—represent one of the most abundant immune populations within the tumor microenvironment and have emerged as critical regulators of tumor progression and metastatic dissemination. Malignant tissues actively exploit macrophage recruitment and differentiation to construct a permissive microenvironment that supports angiogenesis, invasion, vascular entry, and metastatic outgrowth. Clinically, high macrophage infiltration, often assessed by markers such as CD68 and CD163, is associated with aggressive disease and poor outcome. In addition, CD204 (scavenger receptor A)-positive TAMs have been reported to represent a highly immunosuppressive subset associated with tumor progression and unfavorable prognosis. Importantly, TAMs are now understood not merely as correlates of tumor progression, but as rate-limiting effectors of immune suppression, tumor cell migration, and therapy resistance, driven by macrophage-intrinsic signaling programs that are increasingly targetable [1,2].

A major conceptual limitation in the field has been the enduring “M1 versus M2” polarization framework. While useful as an initial model, this binary classification fails to capture the diversity and functional specialization of TAMs observed in breast cancer. Instead, TAMs exist along a continuum of phenotypix and functional states shaped by ontogeny (tissue-resident versus monocyte-derived), spatial localization (including hypoxic, perivascular, and invasive niches), tumor-intrinsic programs, and therapy-induced selective pressures. These context-dependent states integrate metabolic, stromal, and immune signals to generate hybrid macrophage phenotypes that cannot be adequately described by classical polarization. This complexity likely underlies the limited efficacy of macrophage-targeting monotherapies and highlights the need for stratification based on macrophage states rather than abundance alone [2].

**Figure 1:**
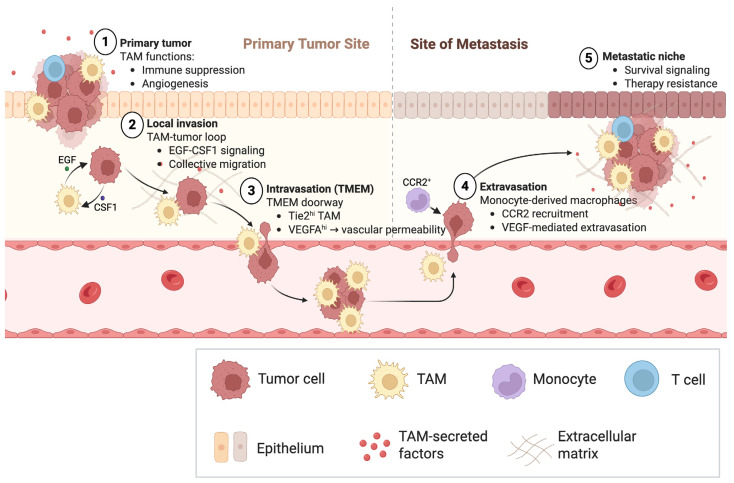
Tumor-associated macrophages coordinate rate-limiting steps of metastatic dissemination.

Here, we propose that TAMs in breast cancer should be understood not as a uniform immunosuppressive population, but as spatially and ontogenetically defined cellular programs that act as rate-limiting enablers of metastatic dissemination and therapy resistance. As summarized in Figure 1, TAMs coordinate sequential steps of metastatic progression—including invasion, Tumor Microenvironment of Metastasis (TMEM)-mediated intravasation, extravasation, and metastatic niche stabilization—through context-specific signaling interactions with tumor cells, vasculature, and immune compartments. Breast cancer provides a uniquely informative model system in which macrophage-dependent invasion, intravasation, and metastatic seeding have been mechanistically validated in vivo and linked to clinically measurable microanatomical structures such as TMEM. By integrating lineage tracing, intravital imaging, single-cell and spatial profiling, and translational studies, this review synthesizes a framework in which specific macrophage states operationalize discrete steps of the metastatic cascade. We further propose that effective macrophage-targeting strategies will require subset-aware, spatially precise, and temporally coordinated interventions rather than global macrophage depletion.

Tumor-associated macrophages (TAMs) regulate multiple stages of the metastatic cascade. In primary tumors, TAMs promote immune suppression and angiogenesis, establishing a permissive microenvironment for tumor progression. During local invasion, reciprocal EGF–CSF1 signaling between tumor cells and macrophages drives coordinated migration toward vasculature. At tumor microenvironment of metastasis (TMEM) doorways, Tie2^hi^/VEGF-A^hi^ perivascular macrophages transiently increase vascular permeability, enabling tumor cell intravasation. At distant sites, CCR2-dependent monocyte recruitment generates metastasis-associated macrophages that facilitate tumor cell extravasation through VEGF-mediated vascular remodeling and adhesive signaling. Within metastatic niches, macrophages provide survival cues and promote therapy resistance, stabilizing metastatic outgrowth. Together, macrophage programs function as spatially organized regulators of dissemination rather than passive bystanders.

## 2. Ontogeny Matters: Monocyte-Derived and Tissue-Resident TAMs

TAMs arise from at least two major sources: circulating inflammatory monocytes that are recruited to tumors and differentiate locally, and embryonically seeded tissue-resident macrophages that persist and can expand within malignant tissues. Evidence for ontogeny-linked specialization comes from lineage-aware mouse models and comparative transcriptional profiling. In pancreatic ductal adenocarcinoma, for example, significant fractions of macrophages originate from embryonic development and expand through in situ proliferation during tumor progression; these embryonically derived TAMs exhibit pro-fibrotic programs, whereas monocyte-derived TAMs display relatively stronger antigen-presentation features [3]. This division has direct therapeutic implications: blocking monocyte recruitment may incompletely deplete macrophage compartments sustained by resident pools, while reprogramming strategies may need to account for origin-specific epigenetic “set points” [3].

A complementary mechanistic advance came from work dissecting the cellular and molecular origin of TAMs in mammary tumors. In this setting, tumor growth induced accumulation of macrophages that were phenotypically and functionally distinct from macrophages in normal mammary tissue. Notably, these TAMs proliferated upon differentiation from inflammatory monocytes, expressed the adhesion molecule VCAM1, and did not display a canonical “alternatively activated” phenotype. TAM terminal differentiation depended on the Notch pathway transcriptional regulator RBPJ; functionally, TAM depletion, but not depletion of macrophages in normal tissue, restored cytotoxic T cell responses and suppressed tumor growth [4]. This study helped formalize a critical principle: tumors generate macrophage states that are developmentally programmed, functionally non-redundant, and therefore targetable [4].

### 2.1. Mapping the TAM “State Space” with Single-Cell and Spatial Approaches

Single-cell transcriptomics has reframed TAM biology from “polarization” to “state space.” In breast cancer, single-cell mapping of immune phenotypes revealed marked heterogeneity across myeloid and lymphoid compartments, identifying multiple macrophage programs co-existing within individual tumors and varying across patients [5]. In non-small-cell lung cancer, tumor-infiltrating myeloid cells demonstrated heterogeneity and dozens of reproducible states across patients, underscoring that macrophages diversify into a spectrum of activation and differentiation programs that cannot be inferred reliably from blood phenotypes [6]. Together, these atlases have enabled two practical moves: first, to define macrophage subsets using multi-gene signatures rather than single markers; and second, to link macrophage states to neighboring cell types, spatial niches and therapeutic context [5,6].

Spatial organization adds another layer of mechanistic meaning. Macrophages positioned in the hypoxic tumor core are exposed to distinct metabolic constraints compared with macrophages in perivascular or stromal compartments. This matters because local conditions do not simply “correlate” with macrophage state: they can instruct it. For instance, hypoxia potently augments macrophage-mediated T cell suppression in vitro in a manner dependent on macrophage expression of HIF-1α, and myeloid HIF-1α promotes tumor progression by sustaining immunosuppressive function [7]. Tumor metabolites can act similarly: lactic acid produced by tumor glycolysis induces VEGF expression and promotes an M2-like polarization program in macrophages through HIF-1α, with lactate-induced arginase 1 contributing functionally to tumor growth [8]. These experiments establish causality for a widely invoked concept: macrophage suppression in tumors is frequently a metabolically enforced phenotype, not merely a cytokine-driven choice [7,8].

In addition to hypoxia and lactate, emerging evidence suggests that iron metabolism represents a critical and underappreciated metabolic axis shaping TAM function and heterogeneity. Iron metabolism has emerged as an important and still underappreciated determinant of tumor-associated macrophage (TAM) function. Beyond their classical immunoregulatory roles, TAMs can actively shape local iron availability through lipocalin-2 (LCN2)-mediated trafficking and ferroportin-dependent export. In breast cancer models, macrophage-derived LCN2 has been associated with tumor onset, lung metastasis, and recurrence, while genetic loss of LCN2 increased iron retention in TAMs and reduced iron accumulation, migration, and proliferation in tumor cells. Mechanistically related observations from other tumor settings further support this concept: macrophage-derived inflammatory signals can induce tumor-cell LCN2/SLC22A17 programs under iron-restricted conditions, and spatial single-cell analyses have identified TAM states consistent with iron-efflux programs, including STAB1^+^ macrophages enriched for SLC40A1/ferroportin and reduced ferritin expression, consistent with a tumor-promoting iron-export phenotype [9,10]. Collectively, these findings indicate that TAMs can function as dynamic regulators of iron distribution within the tumor microenvironment, thereby sustaining malignant cell fitness and progression.

Recent studies also show that iron-related TAM states are not adequately captured by the traditional M1/M2 framework. Heme catabolism through heme oxygenase-1 (HO-1) can define a prometastatic macrophage program associated with immunosuppression, angiogenesis, and epithelial-to-mesenchymal transition, and in breast tumor models HO-1 inhibition during chemotherapy shifted TAMs toward a more inflammatory phenotype and improved CD8^+^ T cell-associated antitumor activity [11]. In parallel, conserved iron-rich TAM subsets (iTAMs) with high intracellular iron and enrichment of heme- and iron-metabolism genes have been shown to support angiogenesis and immunosuppression through Bach1–Ednrb-linked programs [12]. Thus, iron should be considered an organizing axis of TAM heterogeneity in breast cancer, integrating heme breakdown, iron sequestration versus export, immune suppression, vascular remodeling, metastasis, and therapy response, while also highlighting potentially actionable pathways such as HO-1, LCN2, and ferroportin/hepcidin signaling.

### 2.2. TAMs as Suppressors of Anti-Tumor Immunity: From Cytokines to “Cellular Geography”

Mechanistic studies increasingly converge on TAMs as organizers of immune dysfunction through multiple, partially redundant routes. One of the clearest chemotherapy-linked examples involves an IL-10/IL-12 circuit. In breast cancer models, TAM-derived IL-10 indirectly limits chemotherapy efficacy by inhibiting IL-12 production in tumor-associated dendritic cells, thereby blunting CD8^+^ T cell-dependent antitumor responses; IL-10R blockade restores DC IL-12 and improves responses to paclitaxel and carboplatin, and IL12A/cytotoxic effector gene expression in human tumors associates with pathological complete response to paclitaxel [13]. This work highlights an actionable point of control: TAMs can suppress immunity not only by inhibiting T cells directly, but by disarming dendritic cells that are required to sustain effective cytotoxic responses during therapy [13].

Beyond soluble mediators, macrophages can suppress immunity by shaping lymphocyte positioning. In human lung tumor slices and complementary mouse models, stromal macrophages form prolonged contacts with CD8^+^ T cells that reduce T-cell motility and keep lymphocytes sequestered in stromal regions, away from malignant cell nests. CSF1R inhibition disrupts this macrophage-dependent trapping, increases CD8^+^ T-cell motility, and facilitates access to tumor-cell regions, improving response to anti-PD-1 therapy in preclinical models [14]. This “geography of suppression” is clinically resonant because it connects immune exclusion to a tractable intervention that changes lymphocyte distribution, not only activation state [14].

Macrophages can also limit checkpoint therapy via antibody handling. In vivo imaging indicates that anti-PD-1 antibodies bind PD-1^+^ tumor-infiltrating CD8^+^ T cells soon after administration, but this engagement is short-lived: antibody is rapidly removed from the T-cell surface and accumulated by PD-1^−^ tumor-associated macrophages via Fcγ receptor-dependent mechanisms. Blocking Fcγ receptors prolonged anti-PD-1 binding to CD8 T cells and enhanced tumor regression in mice [15]. These data provide a rare, pharmacologically concrete example of how myeloid cells can modulate checkpoint efficacy through Fc–FcγR biology, implying that antibody engineering and myeloid context may be coupled determinants of response [15].

Finally, macrophages themselves can be direct targets of checkpoint pathways. PD-1 can be expressed by mouse and human TAMs, and PD-1^+^ TAMs show reduced phagocytic capacity; in model systems, PD-1/PD-L1 blockade enhances macrophage phagocytosis and limits tumor growth via a macrophage-dependent mechanism [16]. This observation reframes checkpoint blockade as potentially bimodal, acting through both adaptive and innate arms, while also raising mechanistic questions about when macrophage-intrinsic checkpoint signaling dominates clinical behavior [16].

## 3. Transition to Metastasis: Macrophages as Enablers of Movement, Entry and Seeding

The metastatic cascade creates multiple entry points for macrophage control—particularly during invasion and intravasation in the primary tumor, and during extravasation and early survival at distant sites. Intravital imaging and in vivo invasion assays provided early mechanistic evidence that tumor cells and macrophages engage in a cooperative migration program. A paracrine loop in mammary tumors showed that gradients of EGF or CSF-1 stimulate co-collection and co-migration of tumor cells and macrophages despite segregated receptor expression (EGFR on tumor cells; CSF-1R on macrophages), and inhibition of either pathway reduces migration of both cell types [17]. This is a foundational example of how macrophages can act as guides and amplifiers of tumor cell motility in vivo rather than simply secreting generic growth factors [17].

At distant sites, recruitment of inflammatory monocytes and their differentiation into metastasis-associated macrophages can be required for efficient metastatic establishment. In breast cancer metastasis models, interrupting CCL2–CCR2 signaling impairs recruitment of CCR2^+^ inflammatory monocytes to metastatic sites, reduces metastatic burden, and improves survival; mechanistically, these monocytes facilitate tumor cell extravasation in a VEGF-dependent manner [18]. Downstream, CCL2-triggered chemokine cascades within macrophages (including CCL3–CCR1 signaling) enhance retention of metastasis-associated macrophages and promote metastatic seeding, suggesting that distal nodes of the cascade may offer therapeutic leverage with potentially different toxicity profiles compared with upstream blockade [19]. These studies underline a recurring theme: macrophage control of metastasis is often mediated by defined chemokine circuits that can, in principle, be interrupted at multiple points [18,19].

## 4. TAMs in Metastasis: Mechanistic Anchors in Breast Cancer

Metastasis is not a single event but a sequence—local invasion, vascular entry (intravasation), survival in circulation, extravasation, and colonization—and macrophages have experimentally validated roles at multiple steps. In breast cancer, intravital imaging and in vivo invasion assays demonstrate that tumor cells exploit macrophage guidance cues to leave the primary lesion. Wyckoff and colleagues combined an in vivo chemotaxis/invasion assay with intravital imaging to show coordinated recruitment and co-migration of tumor cells and macrophages toward EGF or CSF-1 cues; despite receptor segregation (EGFR on tumor cells; CSF-1R on macrophages), inhibition of either pathway prevent migration of cells, consistent with an EGF–CSF-1 paracrine loop that supports tumor cell motility [17]. This invasion logic generalizes beyond a single ligand pair: heregulin β1 and CXCL12 can trigger invasion that still depends on the EGF/CSF-1 paracrine loop, underscoring that macrophage assistance represents a convergent “final common pathway” for multiple upstream pro-migratory cues [20]. In practical terms, these studies justify treating TAMs as active invasion partners that can set the rate of local dissemination in breast cancer, rather than as downstream markers of inflammation.

## 5. Perivascular TAMs and TMEM Doorways: From Prognostic Microanatomy to Causality

The intravasation bottleneck—where tumor cells cross endothelium into blood—is increasingly understood through the lens of microanatomical intravasation niches. The TMEM was originally defined in human breast cancers as a tri-cellular structure composed of (i) a perivascular macrophage, (ii) a Mena-overexpressing tumor cell, and (iii) an endothelial cell, all in direct contact. In a foundational clinical study, TMEM density in human breast carcinoma samples predicted systemic (hematogenous) metastasis independently of traditional clinicopathologic features [21]. Subsequent work further supported TMEM as a clinically meaningful metric: TMEM score predicted distant metastasis risk in ER^+^/HER2-negative disease, independently of classical variables [22]. These studies established clinical plausibility, but the critical leap to mechanism required direct observation.

Mechanistic causality came from high-resolution intravital imaging showing that brief, focal endothelial permeability bursts—and associated tumor cell entry into vessels—map to TMEM “hotspots,” rather than being distributed diffusely across tumor vasculature. These events depend on perivascular Tie2^hi^/VEGF-A^hi^ macrophages, which locally disrupt endothelial junctions; macrophage depletion reduces TMEM-associated leakiness and circulating tumor cells. Importantly, macrophage-specific VEGFA ablation restored junctional integrity and reduced intravasation—pinning a macrophage-intrinsic VEGFA axis as a mechanistic driver of TMEM function [23]. This provides a rare and powerful example in metastasis biology where (i) a histologic structure is prognostic in humans, and (ii) the same structure is mechanistically validated in vivo with perturbation experiments.

**Figure 2:**
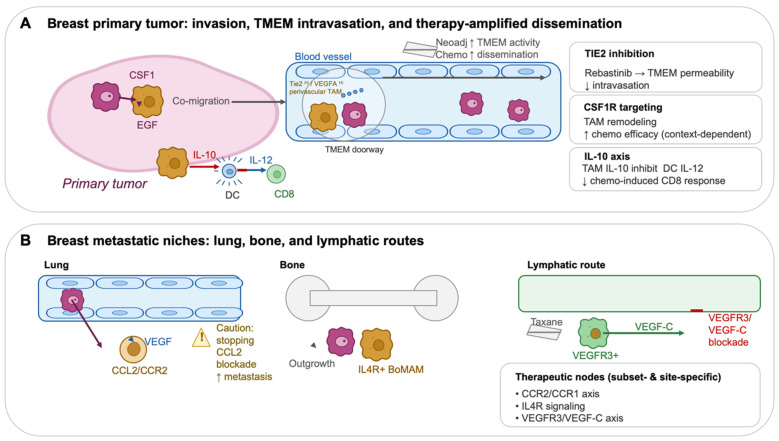
Macrophage-dependent dissemination and therapeutic vulnerabilities in breast cancer. (**A**). Breast primary tumor: invasion, TMEM-mediated intravasation, and therapy-amplified dissemination. Within primary breast tumors, tumor cells and macrophages engage in reciprocal CSF1–EGF signaling that promotes coordinated migration toward blood vessels. The CSF1 arrow indicates tumor cell–derived CSF1 signaling to macrophages, whereas the EGF arrow indicates macrophage-derived EGF signaling back to tumor cells, together supporting tumor cell–macrophage co-migration. The co-migration arrow represents directional movement of tumor cells and macrophages toward the vasculature. At tumor microenvironment of metastasis, or TMEM, doorways, Tie2^hi^/VEGF-A^hi^ perivascular macrophages transiently increase vascular permeability, enabling tumor cells to enter the blood vessel. The arrow from the TMEM doorway into the blood vessel indicates tumor cell intravasation and hematogenous dissemination. The neoadjuvant chemotherapy arrow indicates therapy-associated increases in TMEM activity and tumor cell dissemination. Macrophage-derived IL-10 suppresses dendritic cell IL-12 production, thereby limiting CD8^+^ T cell activation and restraining anti-tumor immunity. In this immune-regulatory pathway, the IL-10 arrow represents macrophage-to-dendritic-cell immunosuppressive signaling, while the IL-12 arrow toward CD8^+^ T cells represents dendritic-cell-mediated T cell activation. Therapeutic interventions targeting Tie2 signaling, CSF1R pathways, or the IL-10 axis are shown as inhibitory nodes that may modulate macrophage function, vascular permeability, dissemination, and immune activation in a context-dependent manner. (**B**). Breast metastatic niches: lung, bone, and lymphatic routes. At distant sites, recruited CCR2-dependent monocytes generate metastasis-supportive macrophages that promote tumor cell survival and outgrowth. In the lung, the CCL2/CCR2 arrow indicates monocyte recruitment and macrophage accumulation within the metastatic niche, while the VEGF arrow indicates macrophage-derived VEGF signaling that promotes vascular remodeling and metastatic seeding. The caution symbol highlights that discontinuation of CCL2 blockade may paradoxically increase metastasis. In bone, the outgrowth arrow represents expansion of metastatic tumor cells supported by IL4R^+^ bone-associated macrophages. Along lymphatic routes, the VEGF-C arrow indicates VEGF-C–mediated signaling toward VEGFR3^+^ lymphatic endothelial cells, promoting lymphatic dissemination. Red inhibitory markings indicate therapeutic blockade of VEGFR3/VEGF-C signaling. Therapeutic nodes targeting CCR2/CCR1 signaling, IL4 pathways, or VEGFR3–VEGF-C interactions highlight subset- and site-specific vulnerabilities in macrophage-driven metastasis. Together, these pathways illustrate how macrophage programs enable metastatic dissemination and create therapeutic opportunities, while also explaining why macrophage-targeted interventions may produce context-dependent outcomes.

A further refinement is that TMEM is coupled to tumor-cell invasive competence. Mena isoform switching has been linked to invasive behavior, with MenaI^NV^ associated with tumor cell discohesion, invasion, and intravasation and correlating with TMEM score in human samples—supporting the notion that TMEM integrates a macrophage vascular-opening program with a tumor-cell motility program [24]. Finally, TMEM biology has implications beyond primary tumors as lymph node metastases can themselves seed distant sites hematogenously, rather than being purely “endpoints” of lymphatic spread [25]. Collectively, TMEM provides a mechanistically grounded framework for how perivascular TAMs can convert local invasion into systemic dissemination as depicted in Figure 2.

## 6. Metastasis-Associated Macrophages (MAMs) in Lung: Recruitment, Retention, and Survival Signaling

Once tumor cells reach the lung microvasculature, macrophage-lineage cells can promote extravasation and early survival. A landmark Nature study demonstrated that tumor-derived CCL2 recruits inflammatory monocytes (CCR2-dependent) that promote breast cancer metastasis; inhibition of CCL2–CCR2 signaling reduced monocyte recruitment, inhibited metastasis, and prolonged survival. Mechanistically, inflammatory monocytes promoted tumor cell extravasation in a process requiring monocyte-derived VEGF [18]. This work links a discrete chemokine axis to a defined metastatic step (extravasation), with in vivo functional outcomes.

Recruitment is only part of the story: macrophages also need to be retained and activated within metastatic foci. Kitamura and colleagues delineated a CCL2-triggered chemokine cascade in macrophages that enhances retention of metastasis-associated macrophages and promotes metastatic seeding, identifying CCR1 as a distal node in this relay with potential therapeutic leverage [19]. At the single-lesion level, direct macrophage–tumor cell physical interactions can be pro-survival. Chen et al. showed that aberrant tumor-cell VCAM-1 expression provides a survival advantage to breast cancer cells in leukocyte-rich microenvironments (notably lung) by tethering metastasis-associated macrophages via α4 integrins (VLA-4) and delivering survival signaling—demonstrating that macrophages can be adhesive niche partners during colonization [26]. In parallel, macrophage-intrinsic receptor signaling can regulate metastatic potency without necessarily controlling macrophage numbers. FLT1 (VEGFR1) signaling in metastasis-associated macrophages promoted an inflammatory gene signature which promoted breast cancer lung metastasis; FLT1 inhibition did not block recruitment but reduced pro-metastatic macrophage programming, with CSF1-mediated autocrine signaling acting downstream to restore tumor-promoting activity [27]. Together, these studies support a multi-layer model: CCL2/CCR2 governs supply, CCR1-linked cascades govern retention/amplification, and adhesion + FLT1/CSF1 signaling governs survival and functional activation within metastatic lesions.

## 7. Tissue-Resident Lung Macrophages: A Distinct Metastatic Niche Axis

Not all macrophage contributions to lung metastasis are mediated by recruited monocytes. Tissue-resident alveolar macrophages can accumulate in premetastatic lungs through local proliferation and can suppress anti-tumor T cell responses. In a breast cancer model, alveolar macrophages promoted lung metastasis by suppressing antitumor T cells and accumulated through complement C5a receptor-mediated proliferation rather than recruitment from circulation [28]. This is a critical nuance for therapeutic logic: strategies that block monocyte recruitment may not sufficiently neutralize resident macrophage programs that expand locally and impose immune suppression.

## 8. Bone Metastasis: IL4R-Dependent BoMAM Programs and Monocyte Origin

Breast cancer bone metastasis is shaped by specialized macrophage programs. Ma and colleagues identified CD204^+^ IL4R^+^ bone metastasis-associated macrophages (BoMAMs) that promote metastatic outgrowth. These BoMAMs were largely derived from CCR2-recruited monocytes (not CD169^+^ resident macrophages in their framework), and monocyte/macrophage-restricted IL4R ablation significantly inhibited bone metastasis growth, supporting IL4R signaling as a mechanistic dependency of a pro-metastatic macrophage subset [29]. This study is especially helpful for a review because it integrates (i) subset identification, (ii) ontogeny inference, and (iii) macrophage-restricted genetic causality in the metastatic site.

Conceptually, the bone site highlights a different mode of macrophage involvement than the lung. Whereas lung metastasis studies emphasize monocyte recruitment, extravasation, and early survival within a vascular niche [18,19,26,27], the bone setting appears to place greater weight on macrophage support of metastatic outgrowth within a highly specialized tissue microenvironment [29]. In this context, IL4R^+^ BoMAMs are notable not only because they define a distinct subset, but because macrophage-restricted IL4R ablation directly limits metastatic growth, indicating that macrophages remain functionally important after tumor cells have already seeded bone [29]. This distinction is therapeutically relevant: in bone disease, effective intervention may require disrupting macrophage-derived trophic and niche-support signals within established lesions, rather than focusing solely on blocking monocyte recruitment. More broadly, the bone metastatic niche reinforces a central theme of TAM biology—macrophage programs are organ-instructed, and mechanisms defined in lung metastasis cannot be assumed to generalize unchanged across tissues.

## 9. Therapy Can “Rewire” Macrophage-Driven Dissemination Pathways

Recent work has emphasized that therapy itself can remodel macrophage states and dissemination routes. In the lymphatic compartment, chemotherapy can create pro-metastatic macrophage programs. Alishekevitz and colleagues showed that macrophages from chemotherapy-treated hosts can, when transferred, induce lymphatic remodeling in otherwise untreated tumors in a VEGFR3-dependent manner, promoting lymphangiogenesis and metastasis via a VEGF-C/VEGFR3 axis [30]. Complementing this, paclitaxel therapy can promote metastatic behavior in breast cancer models through inflammation-linked pathways, with mechanistic emphasis on tumor-intrinsic TLR4 signaling contributing to pro-metastatic effects [31]. These data reinforce a key principle: TAM biology in metastasis is not static—standard therapies can create new macrophage-dependent routes to dissemination and relapse.

## 10. Brain Metastasis: Microglia/Macrophages Can Restrain or Promote Disease, Shaping Therapeutic Strategy

Breast-to-brain metastasis introduces additional complexity because resident microglia and recruited macrophages can have divergent roles. Recent work showed that microglia can promote anti-tumor immunity and suppress breast cancer–brain metastasis, emphasizing that “myeloid depletion” is not uniformly beneficial in the CNS context [32]. Conversely, macrophage-targeted therapies in brain metastasis can evoke adaptive resistance. It has been reported that the CSF2-driven macrophage activation could promote adaptive resistance to CSF1R inhibition in breast-to-brain metastasis, highlighting feedback circuitry that can reconstitute pro-tumor macrophage function despite pathway blockade [33]. For breast cancer reviews, these findings argue for state-aware and compartment-aware myeloid interventions, particularly in the CNS.

These observations suggest that the CNS should not be viewed simply as another metastatic site populated by TAMs, but as tissue in which resident and recruited myeloid compartments coexist under unusually strong anatomical and immunologic constraints. In contrast to the lung, where monocyte recruitment and macrophage retention are prominent organizing principles [18,19], brain metastasis appears to be shaped by a more delicate balance between microglia-mediated tumor restraint and the adaptive reconstitution of tumor-supportive macrophage programs under therapeutic pressure [32,33]. This has important implications for treatment design. Broad myeloid depletion may remove pathogenic macrophage populations, but it may also compromise microglial functions that help sustain anti-tumor immunity, whereas selective pathway blockade can be undermined by compensatory cytokine circuits such as CSF2 [32,33]. Accordingly, the brain metastatic setting may require compartment-aware interventions that distinguish resident microglia from recruited macrophages and prioritize state-specific reprogramming over nonselective depletion.

## 11. Unresolved Questions and Conceptual Tensions in TAM Biology

Despite major mechanistic advances, several conceptual tensions continue to shape the field. These tensions are not all of the same kind and are best understood as arising from three partially overlapping domains: unresolved biological questions, methodological constraints, and translational uncertainties. Some reflect fundamental biological uncertainty, including whether ontogeny imposes durable functional constraints in human tumors or is largely overridden by local microenvironmental cues, whether TAM states represent stable programs or dynamically interconvertible phenotypes, and whether spatially defined macrophage niches such as TMEM represent a general principle of dissemination or a breast cancer-enriched mechanism [3,4,21,22,23]. Others arise primarily from methodological limitations. Much of the strongest evidence for TAM ontogeny and function comes from murine lineage-tracing and perturbation studies, whereas human studies rely predominantly on snapshot-based single-cell and spatial profiling; differences in tissue processing, platform sensitivity, and annotation frameworks complicate cross-study comparisons and make it difficult to distinguish discrete macrophage subsets from continuous state gradients [5,6]. A third set of issues is most relevant to clinical translation: which macrophage programs are truly rate-limiting in human disease, at what stage of progression or therapy they are most actionable, and whether targeting recruitment, survival, or local function will provide durable benefit without disrupting protective tissue-resident macrophages or eliciting compensatory myeloid responses [28,32,33,34,35,36]. Framing the field in this way helps separate discovery priorities from therapeutic decision points and emphasizes that progress will depend not only on defining additional TAM states, but on linking those states to causal function, spatial niche, and treatment context.

## 12. Therapeutic Implications of TAM Biology in Breast Cancer

### 12.1. Breast Cancer: Moving from “TAM Depletion” to “Precision Interception of Dissemination and Resistance Circuits”

A central therapeutic lesson from breast cancer is that TAMs are not only immunosuppressive but can also be mechanistic enablers of dissemination and context-dependent determinants of treatment response. This motivates two complementary strategies: (i) reprogram or deplete macrophage programs that sustain tumor growth and blunt therapy, and (ii) intercept macrophage-dependent “escape routes,” particularly TMEM-mediated intravasation and therapy-induced metastatic phenotypes.

#### 12.1.1. CSF1/CSF1R Axis: Pharmacologic TAM Modulation Is Feasible, but Efficacy Likely Requires Rational Combinations

Preclinical work using selective CSF1R inhibition showed that disrupting macrophage turnover in cervical and mammary tumors can increase intratumoral CD8^+^ T cell infiltration and delay tumor growth, supporting CSF1R as a tractable macrophage dependency [37]. In genetically engineered breast cancer models, CSF1R blockade enhanced the efficacy of platinum chemotherapy by stimulating intratumoral type I interferon signaling and remodeling suppressive myeloid programs, providing a mechanistic basis for combining myeloid targeting with cytotoxic therapy rather than using CSF1R inhibitors as monotherapy [38]. Clinically, CSF1R-directed agents can achieve pharmacodynamic effects on TAMs, but durable single-agent anti-tumor activity has generally been limited; for example, emactuzumab reduced immunosuppressive TAMs but did not yield clinically meaningful activity alone or with paclitaxel in advanced solid tumors, reinforcing the need for biomarker-guided combinations [34]. A phase Ib study combining the CSF1R inhibitor pexidartinib (PLX3397) with weekly paclitaxel demonstrated feasibility and biomarker evidence of CSF1R pathway inhibition but also illustrates the translational challenge of converting macrophage modulation into consistent clinical responses [35].

#### 12.1.2. “Chemo-Induced Dissemination” and TMEM Doorways: A Breast Cancer-Specific Vulnerability with an Actionable Kinase Target

Breast cancer provides unusually direct evidence that some therapies can paradoxically increase the probability of dissemination by activating macrophage-dependent intravasation niches. In models and matched patient samples, neoadjuvant chemotherapy increased TMEM density/activity and circulating tumor cells, linking standard-of-care treatment to a defined microanatomical escape route [39]. This has therapeutic implications because TMEM function is macrophage-driven: perivascular Tie2^hi^/VEGF-A^hi^ macrophages are key effectors of TMEM “doorway” activity, enabling localized vascular permeability and intravasation [23]. Rebastinib (selective TIE2 inhibitor) blocks function and recruitment of Tie2^hi^ macrophages and inhibits TMEM-mediated intravasation, reducing metastasis in mammary carcinoma models [40]. Translationally, a phase Ib clinical and pharmacodynamic study evaluated rebastinib with paclitaxel or eribulin in HER2-negative metastatic breast cancer, explicitly grounded in the TMEM doorway hypothesis [41]. Together, these data support a precision intervention concept: rather than “eliminating macrophages,” target the perivascular macrophage program that operationalizes dissemination and deploy it in contexts (e.g., taxane-based therapy) where TMEM activity may be therapy-amplified [39,40,41].

#### 12.1.3. CCL2/CCR2 Recruitment Blockade: Attractive Biology, but with an Essential Cautionary Signal

Monocyte recruitment via CCL2/CCR2 supports metastatic seeding and extravasation in breast cancer models [18]. However, a critical Nature study demonstrated that cessation of CCL2 inhibition can cause an “overshoot” in metastasis and accelerate death, driven by rebound monocytosis and pro-angiogenic programs—highlighting that transient chemokine blockade may have dangerous withdrawal biology [36]. For an evidence-based therapeutic section, this is worth stating explicitly: CCL2/CCR2 remains mechanistically compelling, but safe clinical translation likely depends on continuous suppression, careful sequencing, or multi-node blockade of the downstream inflammatory/angiogenic cascade rather than short-course use [36].

#### 12.1.4. Therapy-Educated Macrophages and Lymphatic Metastasis: Mitigating Host Response to Taxanes

A distinct, targetable axis is the lymphatic remodeling program induced by chemotherapy-educated macrophages. After paclitaxel, VEGFR3-expressing macrophages promoted lymphangiogenesis and metastasis, and VEGF-C/VEGFR3 blockade reduced this pro-metastatic lymphatic response [30]. This suggests a clinically testable hypothesis in breast cancer: in settings where nodal/lymphatic progression is a dominant risk, combining taxanes with interventions that suppress macrophage-driven lymphangiogenesis (e.g., VEGFR3/VEGF-C axis inhibitors) may reduce therapy-induced dissemination [30].

Key TAM functional programs and candidate intervention nodes discussed in this review are summarized in Table 1.

## 13. Conclusions and Future Directions

The modern understanding of tumor-associated macrophages has shifted from a simplistic “M2 macrophages support tumors” statement to a multidimensional model in which macrophage origin, spatial localization, and microenvironmental cues generate distinct macrophage states with specialized roles in angiogenesis, invasion/intravasation, immune exclusion, metastatic niche formation, and therapy resistance. Mechanistic studies provide causal evidence in model systems that macrophages can be rate-limiting partners in metastatic dissemination [17,20,23], and clinical studies support the prognostic relevance of TMEM in patients [22]; recruitment pathways such as CCL2/CCR2 drive metastatic monocyte influx [18], and macrophage cytokines and microanatomical placement can determine treatment outcomes [13,14]. Translational efforts show that macrophage targeting is feasible in humans and produces meaningful pharmacodynamic changes [50,52,55,60], but clinical success likely requires (i) subset-aware targeting, (ii) combination therapy, and (iii) biomarker-guided patient selection. Future TAM-targeting strategies will be most effective when aligned with tumor ecology: selecting the right macrophage program, in the right tumor compartment, at the right time relative to antigen release and T cell priming.

Future work should prioritize (i) harmonized, spatially resolved definitions of TAM subsets that are comparable across platforms and cohorts; (ii) prospective biomarkers that identify TMEM activity and other dissemination circuits in patients; and (iii) combination strategies that time TAM modulation to the window when antigen release, dendritic-cell priming, and T cell recruitment are most permissive. In particular, site- and context-specific myeloid interventions—rather than global depletion—are likely to be required to reduce metastasis risk while preserving protective tissue-resident myeloid functions.

The next phase of TAM research must transition from descriptive atlases to interventional precision. The central challenge is no longer whether macrophages matter, but which macrophage state, in which spatial niche, at which therapeutic window should be intercepted to prevent dissemination while preserving protective myeloid functions.

## Figures and Tables

**Table 1:** Tumor-associated macrophage programs, representative mechanistic evidence, and translational targeting examples highlighted in this review.

TAM Function/Target	Key Mechanistic Evidence (References)	Translational/Clinical Evidence (References)
Angiogenic switch	[42,43]	—
Pro-angiogenic TEM (Tie2^+^)	[44]	—
Vessel normalization via macrophage reprogramming (HRG)	[45]	—
Invasion/intravasation (EGF–CSF1 loop)	[17,20]	TMEM biomarker concept: [22,25]
Monocyte recruitment to metastasis (CCL2)	[18]	anti-CCL2 carlumab: [46,47]
Rebound metastasis after stopping CCL2 blockade	[36]	—
Chemokine cascade retaining MAMs (CCL3/CCR1)	[19]	—
Metastatic survival signaling (VCAM-1/α4)	[26]	—
FLT1 signaling in MAMs	[27]	—
TAM IL-10 blocks chemo-induced immunity	[9]	—
CSF1R blockade macrophage targeting	[48,49]	anti-CSF1R emactuzumab: [50]
CD40 agonist macrophage reprogramming	[51]	CD40 + CSF1R ± PD-1 trial: CD40 + chemo ± PD-1 in PDAC: [52,53]
TAM barrier to T cells	[14]	—
TAM capture of anti–PD-1 antibody	[15]	—
PD-1 on TAMs reduces phagocytosis	[16]	—
PI3Kγ macrophage switch	[54]	eganelisib trial: [55]
TREM2^+^ suppressive TAMs	[56]	anti-TREM2 preclinical: [57]
CD47-SIRPα checkpoint (phagocytosis + adaptive priming)	[58,59]	anti-CD47 phase I: [60]

Abbreviations: TAM, tumor-associated macrophage; TMEM, tumor microenvironment of metastasis; TEM, Tie2-expressing macrophage; MAM, metastasis-associated macrophage.

## Data Availability

No new data were created or analyzed in this study. Data sharing is not applicable to this article.
